# A Case of Acute Pancreatitis developing after Extracorporeal Shock Wave Lithotripsy

**DOI:** 10.5005/jp-journals-10018-1131

**Published:** 2015-01-06

**Authors:** Vedat Goral, Erkan Sahin, Murat Arslan

**Affiliations:** 1 Department of Gastroenterology, Izmir University School of Medicine, Izmir, Turkey; 2 Department of Radiology, Izmir University School of Medicine, Izmir, Turkey; 3 Department of Urology, Izmir University School of Medicine, Izmir, Turkey

**Keywords:** ESWL, Complication, Acute pancreatitis.

## Abstract

Extracorporeal shock wave lithotripsy (ESWL) is a standard treatment method used for the treatment of renal calculi and upper ureteral calculi. Acute pancreatitis is a serious condition which develops due to multiple etiologic factors and is characterized by autodigestion of the pancreas. A case of acute pancreatitis which developed following ESWL performed for right renal calculi treatment is presented here.

**How to cite this article:**

Goral V, Sahin E, Arslan M. A Case of Acute Pancreatitis developing after Extracorporeal Shock Wave Lithotripsy. Euroasian J Hepato-Gastroenterol 2015;5(1):52-54.

## INTRODUCTION

**Fig. 1:**
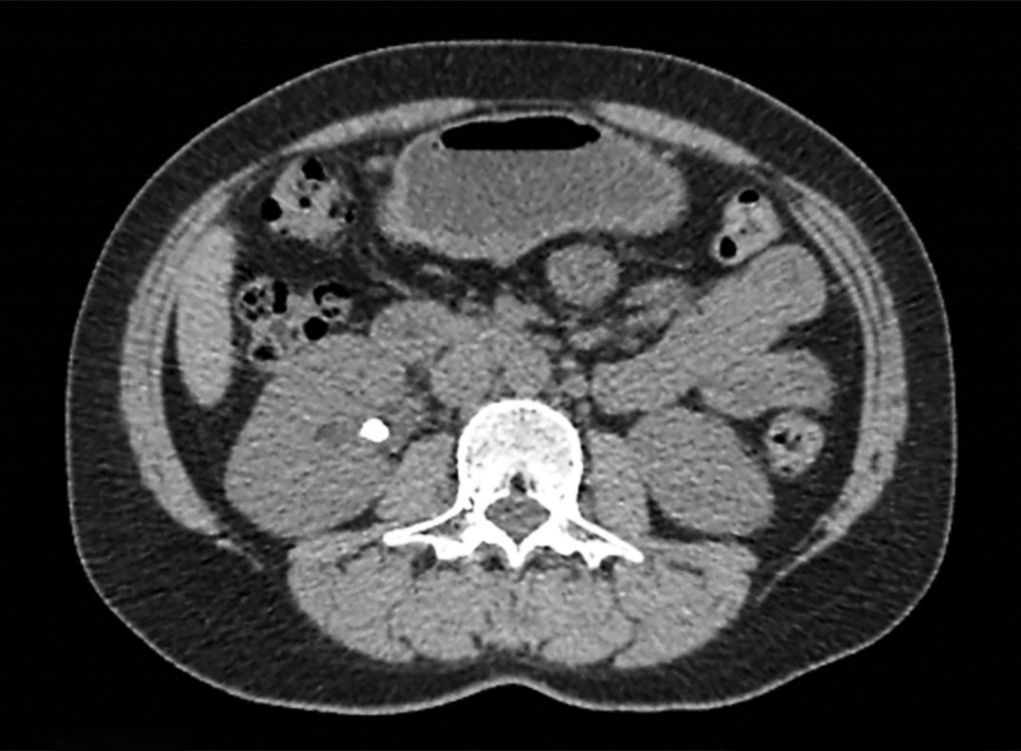
Right renal stone

**Fig. 2:**
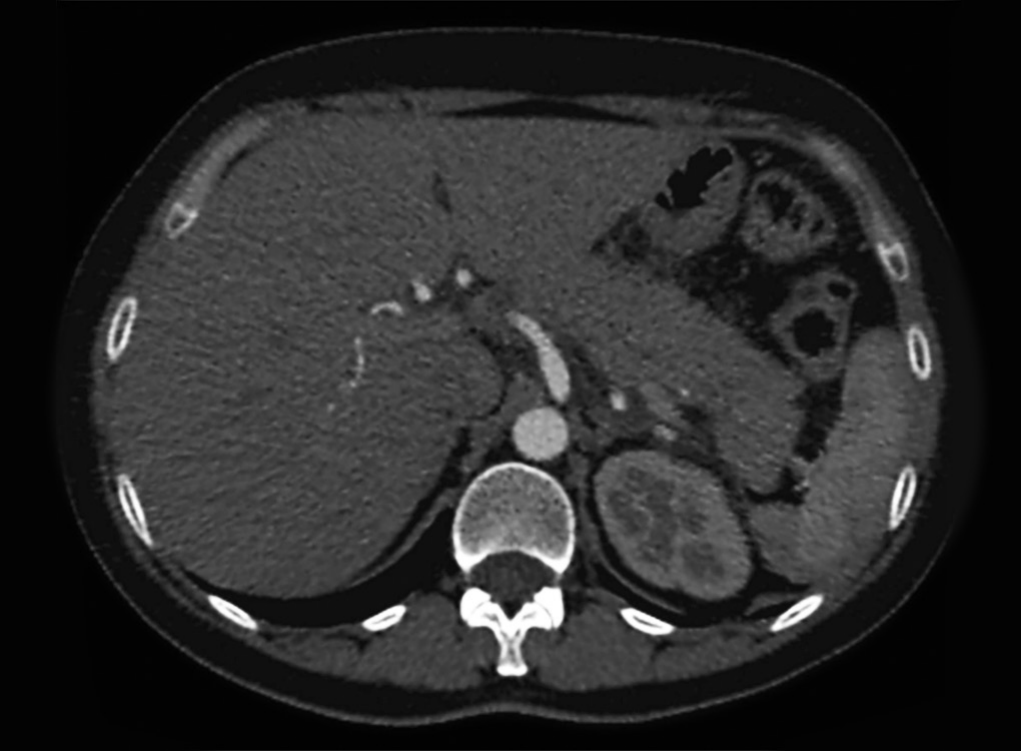
Acute pancreatitis at upper abdominal CT (Balthazar score C)

A 41-year-old female patient had received extracorporeal shock wave lithotripsy (ESWL) at the Urology Department for calculi in the right kidney in our hospital. The ESWL procedure involved 3,000 shots at each session with ELMED (Orlando, FL, USA) lithotripter, starting from 6 voltage and increasing up to 18 to 20 voltage depending on the hardness of the calculus. The patient developed acute pancreatitis after the third session. Upon completion of the third ESWL session, the patient presented to the emergency department with abdominal pain localized in the epigastrium, nausea and vomiting. Acute pancreatitis was diagnosed with the examinations performed, and the patient was, therefore, hospitalized and started conservative treatment. When the patient first presented, she had severe pain and tenderness localized in the epigastrium. Measurements at presentation: amylase: 5178 IU/L (N: 25-125 U/L), lipase: 1030 U/L (N: 10-140 U/L), ALT: 74 IU/ml, AST: 132 IU/ml, WBC: 10300/mm^3^, CRP: 2.7 mgr/dl, and RANSON score was 1 at admission. Patient’s fasting glucose, urea, creatinine, sodium, potassium and calcium values were within normal ranges. In addition, there were 7 mm calculi at the right kidney (mid and inferior calyx) and a 7 mm calculus at the right proximal ureter (Fig. 1). Abdominal computed tomography (CT) revealed acute pancreatitis diagnosis on the basis of volume increases in the pancreas head, corpus and body, contamination of the peripancreatic fatty lanes and minimal peripancreatic fluid (Fig. 2) and Balthazar score was C. Gallbladder and intra/extrahepatic bile ducts were normal. Results of MRCP X-ray were normal. Other causes which may cause acute pancreatitis (e.g. gallstones, alcohol, hypercalcemia, hyperparathyroidism, medications, infections, hypertriglyceridemia) were excluded. Conservative treatment for acute pancreatitis was given at the hospital. Oral intake was stopped except for fluid intake. Follow-up examinations 6 days later demonstrated overall improvement and also improvements in laboratory parameters. The calculus was found to be eliminated at the third session of ESWL.

## DISCUSSION

Acute pancreatitis results from the activation of pancreatic enzymes within the pancreas and autodigestion of the pancreatic tissue.^1^ Although, its etiology differs among countries, gallbladder small stones and alcohol are mainly responsible for about 70 to 80% of the cases. Alcohol ranks first among etiologic factors in western countries while biliary factors are the leading causes in our country. Other causes include abdominal trauma, hypertriglyceridemia, pancreas or ampulla vater tumor, drugs, infectious causes, endoscopic retrograde cholangio-pancreatography (ERCP), autoimmune causes and post-surgical conditions.^1^ Acute pancreatitis has two distinct courses, i.e. mild disease and severe disease. Of the acute pancreatitis cases, 70 to 80% is mild and 20 to 30% is severe. A wide range of clinical manifestations, from mild interstitial edema to severe hemorrhagic gangrene and necrosis may be manifested.

Extracorporeal shock wave lithotripsy is a non-invasive standard method with recent successful use in the treatment of renal calculi.^2,3^ Its complications are mostly transient and mild. There have been occasional reports of potentially life-threatening subcapsular hemorrhage and perirenal hematoma, urosepsis, venous thrombosis, biliary obstruction, intestinal perforation, lung damage, aortic aneurysm rupture, intracranial hemorrhage, pneumonia, cardiac arrhythmia and acute pancreatitis.^4-15^ The incidence of these major complications is less than 1%. The incidence of acute pancreatitis developing post-ESWL has been reported as 0.4 to 0.7% in the literature. Our patient’s medical history did not include gallbladder stones, alcohol or drug use, metabolic causes (e.g. hypercalcemia, hyperparathyroidism), infectious agents (e.g. CMV, EBV, HBV) or any other etiologic factors. Extracorporeal shock wave lithotripsy was considered as etiologic factor for acute pancreatitis in this case report. Few isolated reports of acute pancreatitis following ESWL exist in literature, but the exact mechanism of pancreatitis is not known.^4-15^ Possibly as a result of widening of the focus during ESWL, a part of the pancreas was exposed to extracorporeal shock waves resulting in acute cellular damage (trauma effect) leading to small hematoma, microvascular damage and finally to acute pancreatitis.^5,7,9,10^

## CONCLUSION

In cases of sudden abdominal pain, nausea and vomiting following ESWL, consideration should be given to acute pancreatitis, and ESWL should be discontinued immediately to avoid pancreatic damage in these patients.
